# Fine-scale flight strategies of gulls in urban airflows indicate risk and reward in city living

**DOI:** 10.1098/rstb.2015.0394

**Published:** 2016-09-26

**Authors:** Emily L. C. Shepard, Cara Williamson, Shane P. Windsor

**Affiliations:** 1 Department of Biosciences, Swansea University, Swansea SA2 8PP, UK; 2 Department of Aerospace Engineering, University of Bristol, Bristol BS8 1TR, UK

**Keywords:** urbanization, energy landscape, flight, soaring, UAV, gull

## Abstract

Birds modulate their flight paths in relation to regional and global airflows in order to reduce their travel costs. Birds should also respond to fine-scale airflows, although the incidence and value of this remains largely unknown. We resolved the three-dimensional trajectories of gulls flying along a built-up coastline, and used computational fluid dynamic models to examine how gulls reacted to airflows around buildings. Birds systematically altered their flight trajectories with wind conditions to exploit updraughts over features as small as a row of low-rise buildings. This provides the first evidence that human activities can change patterns of space-use in flying birds by altering the profitability of the airscape. At finer scales still, gulls varied their position to select a narrow range of updraught values, rather than exploiting the strongest updraughts available, and their precise positions were consistent with a strategy to increase their velocity control in gusty conditions. Ultimately, strategies such as these could help unmanned aerial vehicles negotiate complex airflows. Overall, airflows around fine-scale features have profound implications for flight control and energy use, and consideration of this could lead to a paradigm-shift in the way ecologists view the urban environment.

This article is part of the themed issue ‘Moving in a moving medium: new perspectives on flight’.

## 1. Introduction

Air is a highly dynamic medium, with flow vectors varying in time and space. At large scales, birds from migrating passerines to vultures vary their flight times and routes in relation to flow vectors [1–4]. This enables many species to reduce their travel costs by exploiting tailwinds or extracting energy from wind gradients and updraughts [5–7]. Even at fine scales, individuals should be able accrue substantial energy savings through their selection of flight route [8,9]. In the terrestrial environment, the complexity of the substrate introduces substantial variability in the airflows above it [10]. Consequently, birds should experience dramatically different flow conditions, and therefore flight costs, with even minor changes in flight trajectory, e.g. opting to fly to one side or another of a ridge, building or tree-line [11]. However, information on how birds respond to airflows at fine scales is lacking. This is probably due to the difficulties of resolving bird flight trajectories [12] in relation to small-scale features (i.e. within metres), as well as establishing how the features themselves modify airflow characteristics. Nonetheless, understanding how birds respond to airflows at these scales is important, not least because the aerial environment is changing [13,14]. Anthropogenic land-use change is affecting substrate characteristics and hence both wind and heat-driven flows [10,15]. More specifically, the construction of buildings and other infrastructure, for example, affects the way that air flows around them.

This study examined the extent to which birds modulate their flight paths in relation to the availability of wind-driven updraughts in an urban environment (Swansea City). We establish a simple experimental scenario, which exploits the fact that birds frequently fly over buildings and trees that border the sea, to investigate space-use at two nested scales. First, we assess whether birds vary their flight trajectories with wind conditions, with the predictions being that (i) birds are more likely to fly along the seafront when onshore winds generate orographic updraughts, i.e. as buildings deflect air upwards, and that (ii) use of this flight path is therefore associated with soaring flight. Second, we investigate the factors that determine the precise, three-dimensional trajectories of birds soaring along buildings, with the expectation that birds will select particular flow characteristics within the region where air is rising. Assuming that birds use these updraughts to maintain, rather than increase, their altitude, the strength of the vertical flow they select will determine their forward speed. Birds can maximize their speed by selecting the strongest vertical flows, but such a strategy would require them to fly close to the buildings, which may increase collision risk. We combine high-resolution data on flight paths with computational fluid dynamics (CFD) modelling to (iii) quantify the flow characteristics selected by birds according to their fine-scale position and (iv) compare measurements of actual flight speed with those that are potentially achievable. Taken together, this will provide insight into the factors affecting the fine-scale flight trajectories of soaring birds.

Gulls are used as model species as they are facultative soaring birds that exploit sources of rising air but also frequently employ flapping flight [16]. Their flight characteristics, including area use, flight mode, airspeeds and climb rates, should therefore provide insight into the energetic consequences of flight in urban environments. While it is well known that urban airflows are particularly complex [17], to date there has been no research into the implications of this for birds flying in this environment. Operating in this environment also represents a potential challenge for small unmanned aerial vehicles (UAVs) [18]. Consequently, the strategies used by birds could prove valuable for UAV flight planning.

## 2. Material and methods

Swansea City is situated on a large bay that is bordered by stretches of buildings or trees (electronic supplementary material, figure S1). In periods with onshore winds (those with a south-easterly component), the air moves over a relatively flat sand beach or water (depending on tide) before it meets the trees or buildings, which deflect the air upwards. Importantly, under these wind conditions, air meeting the bay has not been modified by obstacles upstream. This therefore provides an opportunity to quantify how birds modulate their flight parameters over fine-scale features, in scenarios with relatively simple airflows. Flight data were collected from both herring gulls *Larus argentatus* (HG) and lesser black-backed gulls *L. fuscus* (LBB) and pooled owing to the similarity in their morphology and predicted flight performance.

### (a) Wind conditions and area use

In order to examine the scale at which birds modulate their movements with respect to the wind, the numbers of HG and LBB gulls flying through a single target area were recorded under a range of wind conditions. Surveys were conducted in the 20 min prior to sunset, when large numbers of birds fly to their roost or pre-roost sites during the non-breeding season. The observer was positioned in front of a line of seafront hotels (electronic supplementary material, figure S1) and any gulls that entered a volume of air, defined by the 500 m length of hotels and extending out towards the sea for approximately 100 m, were recorded. Gulls entering this area at any altitude were continually scored according to whether they used flapping or gliding flight, and whether they flew along the hotel line or had a different trajectory. Wind speed and direction were measured with a handheld anemometer at the beginning and end of the session and the means of these values were recorded as the wind speed for a given session. Surveys were conducted over 31 days.

### (b) Deriving flight tracks and airspeed

On days when the wind had a south-easterly component, detailed information on flight characteristics was collected for gulls gliding along the seafront using an Ornithodolite [19]. These surveys were carried out during the day, i.e. earlier in the day than sunset observations. The Ornithodolite is based on a pair of binoculars with an inbuilt laser-rangefinder, compass and inclinometer (Vectronix Vector Aero 21) that enable an observer to record the *XYZ* coordinates of a target (for details see [19]). A series of coordinates for a given bird in flight can be used to record the flight path (hereafter termed an individual run or track) and estimate groundspeed. The refresh rate of the vector means that fixes can be obtained at intervals of 2 s or longer.

The system was coupled with a Gill Windsonic anemometer mounted on a 5 m mast, to provide simultaneous measurements of the horizontal wind vector in an unobstructed location [19]. Helium balloons were also released and tracked with the Ornithodolite, to quantify how the wind vector changed with altitude [19]. Balloons were tracked at least twice per recording session, providing a vertical wind profile every 30 min on average. The raw *XYZ* coordinates for all runs were combined with estimates of wind speed at the flight height of the bird, based on the balloon profiles. This allowed us to estimate true airspeed, the bird's heading [20] and the absolute value of wind support and the cross-wind component (relative to the bird's heading) [21]. Estimates of true airspeed were converted to equivalent airspeed (the airspeed at an air density equivalent to that at standard sea level) to allow comparison of flight speeds between runs [20].

### (c) Modelling airflows

We used a CFD model to examine the relationship between bird flight paths and airflow characteristics for birds gliding over the hotels (see the electronic supplementary material for full details). This required a digital elevation model (DEM) of the observation site, which was built using terrain data (a 2 m resolution LiDAR dataset [22]) and Ordinance Survey building data [23,24] (see the electronic supplementary material for details). The gull flight tracks were imported into the same coordinate system as the DEM. This allowed us to calculate the mean radial distance from the bird to the buildings per run, as well as the angle between the bird and the buildings (flight parallel to, and directly in front of, the roof line, was taken as 0°, increasing to 90° when a bird flew directly above it (electronic supplementary material, figure S2)).

Wind field data were generated using the CFD model in the Quick Urban & Industrial Complex (QUIC) fast response dispersion modelling software [25], using the vertical wind profiles from the balloon releases as the input boundary conditions. QUIC is designed to give relatively fast, yet accurate, wind fields in urban areas, and has been extensively validated against wind tunnel models and experimental urban wind field measurements [26]. A 300 × 300 × 100 m [*X* × *Y* × *Z*] grid was used, with 1 m resolution.

The QUIC model was used to estimate the vertical component of the airflow (the ‘*w*’ component) associated with the three-dimensional position of a bird. This was achieved by averaging *w* values parallel to the buildings, giving a two-dimensional map of the available *w* at each grid position, and extracting the associated values for each gull track. Values of *w* were strongest in the region closest to the windward edge of the hotel roof. To compare the difference between the *w* values selected by the gulls and the highest *w* value available, we extracted the *w* value in the 1 × 1 m grid cell closest to the windward edge of the building.

The outputs of the QUIC model were also used to map the airspeeds that birds could theoretically use to fly along the hotels according to their position. Here, the average *w* values along the axis parallel to the hotel front were converted to estimates of flight speed, assuming that birds matched their sink rates to the *w* component in a manner determined by a fixed-wing glide polar (adapted from the freeware ‘Flight’ [20] and the morphological measurements in the associated database; see http://www.bristol.ac.uk/biology/people/colin-j-pennycuick/research.html). This produced a velocity map of the feasible true airspeeds (see the electronic supplementary material for estimation of model validity).

### (d) Statistical analysis

#### (i) Space-use and flight type in relation to wind conditions

We used R Statistical Software (v. 3.2.2) (R Core [27]) for all statistical analyses and the significance level was set at *α* = 0.05. Generalized additive models (GAMs) were used to analyse the effect of wind direction and strength on the number of gulls observed flying through the target area. GAMs were selected as they allowed us to model a nonlinear relationship without imposing a parametric form. Three different GAMs were fitted in order to examine variation in (i) the total number of LBB and HG flying through the observation area (i.e. pooling observations from flapping and gliding birds), (ii) the number of gliding birds, and (iii) the number of birds gliding along the line of the hotels, as opposed to flying in any other direction. All three GAMs modelled the number of birds as a function of wind direction and wind speed. In model (iii), we also distinguished between birds according to whether or not their trajectory was aligned with the hotels (where the two flight types were given as factors). In this model, the observations from each day were therefore split into two categories. A Poisson family distribution was used to model all bird counts, and we included the hours sampled as an offset on the log-scale to control for differences in sampling effort between days. GAMs were fitted using the mgcv package (v. 1.8–7), and we followed Wood [28] for model fitting and diagnostic tests (see the electronic supplementary material for details).

#### (ii) Variation in airspeed and fine-scale position during soaring flight

Linear-mixed effects models (LMMs) were used to analyse the variation in airspeed and climb rate for gulls gliding along the seafront. LMMs were performed using the R package nlme. In the model of airspeed, wind support, cross-wind component and site (specified as either trees or hotels) were included as fixed effects. Individual run was included as a random variable to account for non-independency of values collected within the same run, and run was nested within observation day. A further LMM was performed to examine variation in climb rate as a function of airspeed, site and the interaction between these variables. Airspeed was included as a fixed effect and run and day were listed as random effects. The R library MASS was used to ascertain whether the response variables required transformations and we checked model assumptions using qqplots (to assess normality of variance) and plots of residuals against fitted values and individual sites (for homogeneity of variance).

A Pearson's correlation was used to examine whether the *w* values selected by birds were correlated with the maximum *w* values available. Linear regressions were used to assess whether the distance and angle to the hotels varied with wind speed.

## 3. Results

### (a) Wind conditions and area use

Surveys of gulls flying through the study area yielded a total of 3650 observations (giving a daily mean of 118 observations ± 81 s.d., range 25–315). While most observation sessions lasted for 20 min, four were cut short owing to the onset of rain. The total number of birds observed per session varied as a complex function of wind strength and direction. Both these variables were strongly significant in explaining gull numbers, as was a two-way interaction between them (*p* < 0.001 in all cases, with the overall model explaining 77.4% of the deviance). The number of flying birds was greatest with easterly wind directions from 50 to 150° and lowest with winds from 210 to 310°. Fewer birds were observed in strong winds (more than 8 ms^−1^), except for wind directions between 100 and 200° (which had the highest number of birds with winds more than 8 ms^−1^).

**Figure 1.**
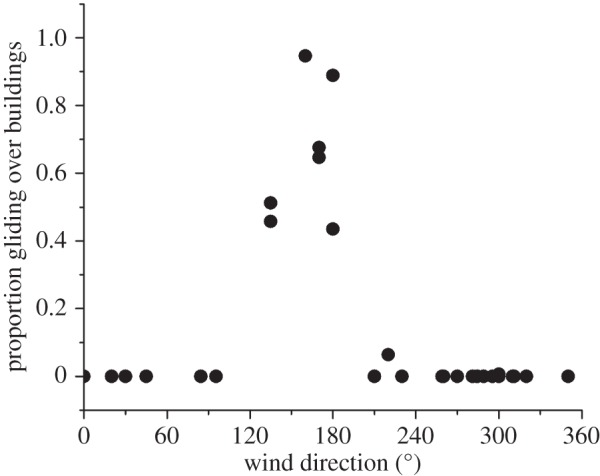
The proportion of birds gliding over the hotel site as a function of the total number of birds observed per session is given in relation to the wind direction for that day. There was a clear peak in gull numbers when model predictions indicated maximum availability of orographic updraughts (winds of around 150°).

The total number of gliding birds showed a general increase with wind strength and also varied with wind direction. The model with wind strength, direction and their interaction explained an estimated 96.5% of the deviance in the numbers of gliding birds, with *p* < 0.001 for each of the model terms (*n* = 31 days). The number of birds gliding along the line of hotels (as opposed to the total number flying through the target area) varied from 0 to 198 per observation session. There was a clear relationship between the number of birds using the hotels and the wind direction, (*p* < 0.001, *n* = 60) (figure 1), with a well-defined peak in wind directions around 150°, and hence with winds that were perpendicular to the front face of the buildings.

### (b) Airspeed and climb rate in gliding birds

Overall, 163 tracks were collected from birds gliding above the hotels (*n* = 102) and trees (*n* = 61) that border Swansea Bay (electronic supplementary material, figure S1). Data were collected when the wind had a south-easterly component and wind speeds varied from 1.9 to 12.4 ms^−1^.

Mean airspeeds across runs ranged from 8.1 to 19.9 ms^−1^ (mean ± s.d. = 13.7 ± 2.48 ms^−1^). The strongest predictors of airspeed were the cross-wind component (*t* = 6.52, *p* < 0.001, d.f. 794) and wind support (*t* = −7.61, *p* < 0.001, d.f. 794), with birds increasing their airspeed in relation to the former and decreasing it in relation to the latter. There was no evidence to suggest these relationships were nonlinear. Airspeed did not vary between flights over trees or buildings (*t* = −1.40, *p* = 0.163, d.f. 163).

Overall, climb rates were low (mean across runs ± s.d. = 0.12 ± 0.36 ms^−1^). Nonetheless, a Wilcoxon signed ranks test showed that the median climb rate was greater than zero (*Z* = 2.05, *p* = 0.040). Climb rate was significantly predicted by airspeed (*t* = −2.24, *p* = 0.025, d.f. 795), but it did not vary between flights over trees and hotels, either as a single factor (*t* = −0.33, *p* = 0.743) or in interaction with airspeed (*t* = −0.29, *p* = 0.770).

### (c) Fine-scale position and airflow selection

**Figure 2.**
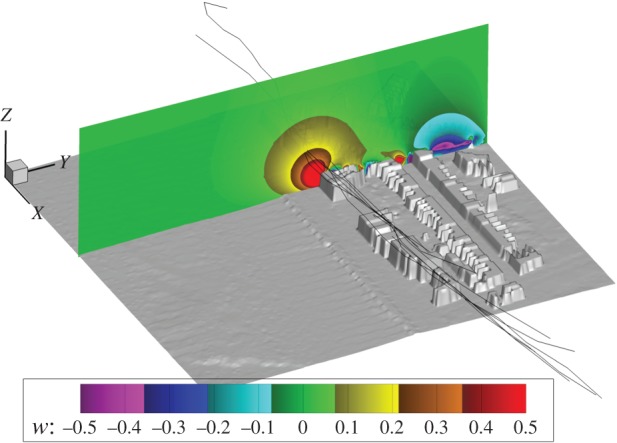
The CFD model output in relation to the digital profile of the hotel site, with the strength of the vertical wind vector component (*w*, in ms^−1^) indicated in colour. The input conditions were a wind speed of 6.7 ms^−1^ and direction of 132° from North, and the tide height was −3.09 m Ordnance Datum Newlyn (ODN). The bird flight paths associated with these wind conditions are indicated by black lines.

Data on bird flight paths and flight speeds were collected on days with a southerly wind component. On these days, there was a mean angle of 34° between the wind and the line of the hotels (range 18–49°) and the mean wind strength was 5.7 ms^−1^ (range 2.2–9.3 ms^−1^). Of those flight paths that fell within the model area, mean *w* values (estimated for each individual track) were not correlated with the maximum *w* values available (Pearson's correlation *n* = 96, *r* = 0.02, *p* = 0.843; figure 2). In fact, the *w* values selected by the birds fell within a reasonably limited range (mean ± s.d. = 0.67 ± 0.20 ms^−1^, 95% CI = 0.63–0.71 ms^−1^).

**Figure 3.**
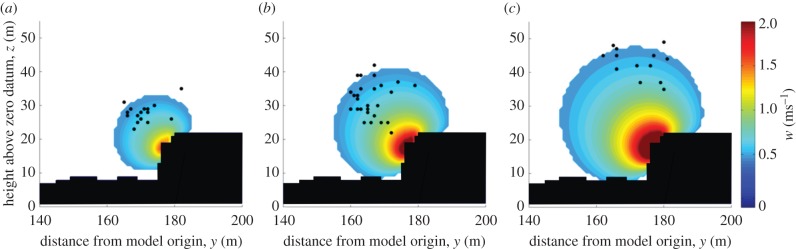
The distribution of the vertical wind vector component (*w*) is given in relation to the profile of the buildings for three cross-wind conditions. The cross-wind strengths were 3.4, 5.5 and 7.6 ms^−1^, moving from left to right. The strength of *w* is illustrated with the colour scale. Round symbols indicate the mean positions of birds flying in each set of conditions.

**Figure 4.**
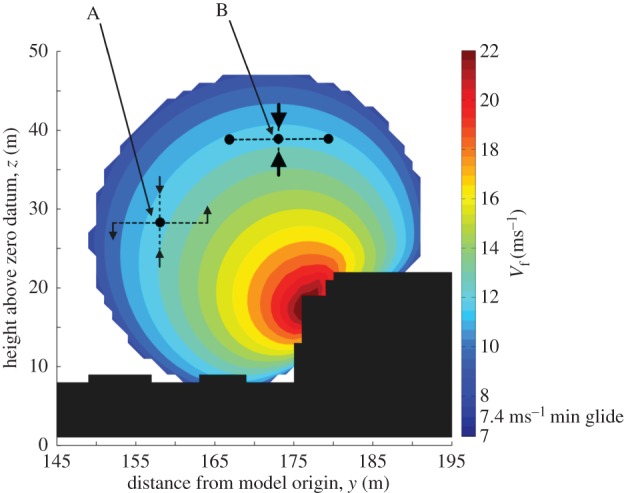
A velocity contour map in relation to flight over the hotels. Predicted airspeeds (in ms^−1^) of birds flying parallel to the hotels are indicated in colour. Two possible positions (A and B) are given for flight at 12 ms^−1^, with dashed lines representing horizontal and vertical displacements from an equilibrium position and black arrows representing the change in lift force produced if the bird does not make any corrective actions. Position A demonstrates the wide velocity range available for a given horizontal displacement for birds flying at relatively low angles. The velocity range available for the same horizontal displacement is much lower at position B, while for vertical displacements the stabilizing forces are stronger at B than A. The input wind conditions are 9.34 ms^−1^ with a direction of 137° from North and a tide height of 1.14 m ODN.

Birds were found to increase their radial distance to the hotels with wind speed (*y* = 0.20 × wind strength + 1.87, *r*^2^ = 0.35, *p* < 0.001). The angle between the position of the birds and the hotels also increased with wind strength (*y* = 0.05 × wind strength + 1.49, *r*^2^ = 0.17, *p* < 0.001; figure 3). The implications of changes in angle were assessed using a map of feasible airspeeds. The quasi-circular contours demonstrated that birds would have been able to maintain a given airspeed when flying at a wide range of angles (figure 4). However, the relative horizontal and vertical widths of a given velocity contour changed with flight angle.

## 4. Discussion

It has been known for some time that birds modulate their movements in relation to airflows at large scales in order to reduce their flight costs [3,5,9,29]. The general context for such studies has been migration; a spectacular, and energetically onerous, annual event. Here, we combine novel, high-resolution data on bird movements with high-resolution models of airflow to demonstrate that birds vary their flight paths in relation to the distribution of updraughts at fine scales in both space and time. While less spectacular than mass migratory movements, the substantial annual time dedicated to ‘the norm’ means that cumulative energy savings derived from judicious area use and flight path selection may be substantial (cf. [30]), particularly for facultative soaring birds. Consequently, even apparently ‘small’ features, such as individual tree-lines or buildings may profoundly affect the daily energy budgets of birds [11,31].

This provides new emphasis to previous findings that small, man-made features can apparently serve to provide energetic benefits to flying birds (e.g. observations of vultures soaring over power plants [32]). Here, we show that birds actually alter their flight paths according to the wind conditions, in order to exploit updraughts generated by such small features (cf. [31]), which has consequences for the many attendant factors linked to space-use (such as predation pressure). In our case, it also provides the first evidence that human activities may change patterns of space-use in flying birds by altering the profitability of the airscape. While changing patterns of land-use have been widely documented, little has been said (in the biological literature) about how these changes may alter patterns of airflow over the land [13,14]. Given we know that the construction and characteristics of buildings, particularly building height, profoundly affects airflows [10], including the distribution of uplift in three dimensions [33], we should expect the ecology of birds that might, or do, use such spaces to be similarly affected.

### (a) Currencies in soaring flight

The relative value of small-scale features to flying animals will depend not only on the way that they modify airflows, but also on the proximate goal of the animal. In this study, there was evidence that birds used orographic updraughts over buildings to commute to their roost or pre-roost sites. An interesting question arising from the work is whether birds vary their roost site in relation to wind conditions, and if so, the consequences this may have for the selection of foraging grounds the following day. Birds also appeared to use the lines of buildings and trees to travel within a foraging patch (here Swansea city centre), as indicated by their flight characteristics (see below). Soaring may offer advantages to remaining stationary, as it enables birds to search new ground for food at low cost [34] and respond quickly when food becomes available. The strength of orographic updraughts declines rapidly with altitude (figure 2), which explains why birds in this study did not use these updraughts to gain height and glide to other areas, as in the case of thermal updraughts [35]. Instead, the value of orographic updraughts in urban environments is that they are predictable (in relation to wind conditions) and persistent, allowing low cost travel within/through a habitat that is associated with other resources (see below).

Although gulls were apparently using the orographic updraughts to travel, the particular flow conditions they selected over the buildings showed they were not maximizing their glide speed. In line with findings from other studies, birds did increase their airspeed with the strength of the headwind and cross-wind components (e.g. [21]). However, they could have increased their airspeed in all conditions by selecting the maximum values of *w* available (figures 3 and 4). For instance, the maximum value of *w* estimated in this study was 2.5 ms^−1^. If birds had flown in this area and matched their sink rate to the rate at which air was rising, they would have achieved airspeeds of 24 ms^−1^. Yet the mean airspeed for birds flying in these particular conditions was 13.7 ms^−1^; some 10 ms^−1^ slower than the estimated maximum. It may be that birds in this study did not optimize their flight speed because they were not subject to time constraints (though see also [36]), as data were collected during the non-breeding season and when birds appeared to be flying to maintain station in a foraging patch. This seems likely as there was no relationship between the *w* values that gulls selected and the maxima available. Instead, gulls appeared to modulate their distance from the buildings in order to use a limited range of the available *w*.

Our data suggest that the birds' precise positioning in flight could also be influenced by the need to maintain flight control. While the distance to the buildings may reflect preference for particular *w* values (and the corresponding airspeed), this cannot explain the change in angle, as the distribution of any given *w* value is described by a quasi-circular contour around the buildings (figure 3). One possible explanation could lie with the ease of flight control when subject to wind gusts. Our results suggest that flight control requirements may be reduced when the birds fly at higher angles (figure 4). A horizontal displacement at low angles relative to the wind field would move the bird through a greater range of *w* values than the same displacement at higher angles, requiring the birds to make larger changes to their airspeed in order to maintain their height. Vertical displacements at high angles would appear to be self-stabilizing, with decreases in height leading to increased uplift—which would act to increase the height of the bird—and increases in height leading to decreased uplift, which would act to decrease height. The strength of this self-stabilizing effect is reduced at lower angles relative to the buildings. As the birds stay in a relatively narrow band of *w* values, the relative amplitude of gusts is likely to increase as the mean wind speed increases, which may indicate why birds fly at higher angles with increased cross-wind strength. Nonetheless, what is not clear is why birds did not select high angles at all wind speeds.

Loss of control during flight is likely to be particularly important for birds operating at low altitudes and in cluttered environments. In this sense, exploiting orographic lift over urban features may be a high-risk strategy, as birds must fly relatively close to the substrate in order to benefit from the updraughts. This could also explain, in part, why birds in this study did not exploit the strongest updraughts available, which would have involved flying very close to the buildings at high speed. Loss of flight control in this region is more likely to lead to a collision.

Flight control at low altitudes in gusty urban environments is a significant challenge for UAV operations [18]. Small-scale fixed-wing UAVs are much more strongly affected by gusts and turbulence that larger aircraft, as the wind velocity is comparable with their airspeed [37]. Flying at low altitudes in the highly complex flow field of urban environments, in close proximity to terrain and buildings, is a significant challenge that most current autonomous flight control systems have not been developed to cope with. As such, examining how birds of a similar size and weight to small UAVs overcome these challenges could help to inform UAV flight path planning and flight control system development for flight in the same environments. Low-altitude flight is of interest to UAV engineers as orographic soaring could greatly extend UAV endurance. There has been work on this at larger geographical scales [38,39], and there is great potential for also developing this strategy for UAV flight at the scale of urban operations [33]. Here again, understanding how birds use orographic soaring in these environments could significantly benefit the development of UAV systems facing similar challenges.

### (b) Implications of urban airflows for avian ecology

**Figure 5.**
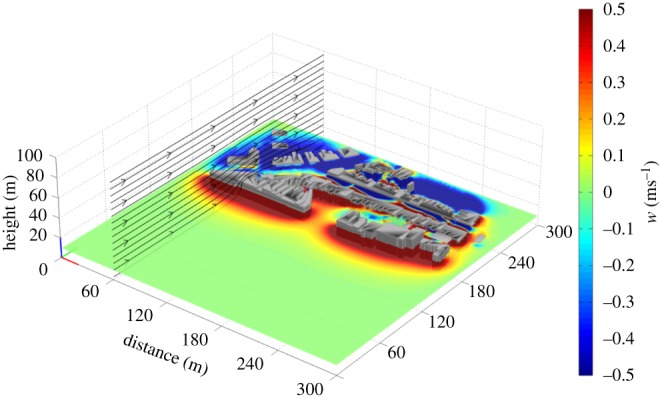
The vertical wind vector component (*w*, indicated with a colour scale) within the study site, at a height of 10 m above ground level and with a wind input of 7 ms^−1^ and a direction of 141°. This illustrates the variability of the flow field and the volume of the downdraughts in relation to the updraughts. The colour scale has been limited to ±0.5 ms^−1^ (just above the minimum sink rate) to highlight the area available for gliding flight at a sustained altitude.

Overall, urban environments are likely to be highly productive in terms of generating wind-driven updraughts (figure 5) [33]. To date, however, this form of energy has not been considered in studies examining the effects of urbanization on wildlife [40]. For gulls, it can be argued that buildings are functionally equivalent to cliffs, in the sense that both are solid vertical features that border the sea and provide updraughts in onshore winds. In erecting lines of buildings along the seafront, urban planning authorities have therefore created features that gulls are predisposed to exploit. The opportunities for low cost movement, coupled with the increased availability of food for generalists such as gulls [41], probably mean that urban habitats (coastal or otherwise) are associated with high net rates of energy gain. All other factors being equal, we would therefore predict increased occupancy and/or exploitation of the urban environment by soaring birds such as gulls. Indeed, while the rise in urban gull populations has been largely attributed to the availability of food resources and nesting habitat [41], it is possible that low movement costs may also have contributed to the success of these populations (cf. [30]).

While on the one hand, the urban environment may be considered ‘profitable’ for soaring flight, it is also likely to entail risks. Even in our very simple study scenario, where relatively uninterrupted airflows met a line of buildings, birds modulated their behaviour in line with a strategy to improve their flight control. Flow regimes in urban environments will be dramatically affected by adjacent rows of buildings, as flows around buildings will interact, producing a highly complex pattern of horizontal and vertical vector components (figure 5). Consequently, there are many interesting questions about the challenges of flying in the urban environment and how animals respond to them, if indeed they can (cf. [18]). For example, it may be that a high power margin (and therefore smaller body size) is advantageous, because birds can respond effectively to variable or risky flows by increasing their speed or switching to climbing flight [20]. Does that mean then, that bird species composition within urban spaces may be modulated by the nature of the buildings, the way buildings are situated with respect to each other and the wind regime? Certainly, Taylor & Thomas [42] have recently demonstrated how morphological optimization criteria in birds vary according to the type of airflow being exploited.

In conclusion, we use high-resolution data on flight paths and airflow models to provide new insight into how birds use the urban environment. Combining different techniques, we show that birds modulate their movement paths to exploit updraughts over features as apparently small as buildings. The availability of updraughts over buildings means that birds such as gulls should be able to experience high rates of net energy gain in urban environments. Indeed, the way that buildings influence airflows may have profound implications for the ecology of a range of species that operate around them. At fine scales, the positioning of soaring birds was consistent with a strategy to ease flight control requirements in unsteady conditions. This demonstrates how the behavioural strategies of flying animals can be used to inform flight planning in UAVs, highlighting ways of reconciling the different currencies of energy gain and risk mitigation. Equally, the ornithological community stands to gain important insights from UAV engineers, such as how the power requirements of flight vary in different flow conditions. Overall, this study represents an early example of what is likely to become an active field of research into flight in complex aerial environments that draws on the fields of ecology and aeronautical engineering.

## Supplementary Material

Supplementary Methods and Results

## Data accessibility

Data on gull flight characteristics (positions, airspeed, climb rate, etc.) are available through from the Dryad Digital Repository: http://dx.doi.org/10.5061/dryad.
