# Supplementary material for: Fine-scale flight strategies of gulls in urban airflows indicate risk and reward in city living
Source: Philos Trans R Soc Lond B Biol Sci. 2016 Sep 26;371(1704):20150394. doi: 10.1098/rstb.2015.0394 (PMC4992718; doi:10.1098/rstb.2015.0394)
Supplement: Supplementary Methods and Results [file rstb20150394supp1.docx]

**Fine scale flight strategies of gulls in urban airflows indicate risk and reward in city living**

**Methods**

***Study location***

Swansea is a coastal city in Wales (figure S1). Ornithodolite data were collected at two sites, evident in figure S1 as lines of gull locations, with the uppermost line of gull locations being the hotel site.

Figure S1. a) The geographical location of Swansea within Wales is indicated by the white square. Swansea bay is indicated in b) with locations of gulls soaring along the seafront given in yellow. Images were generated using GoogleEarth.


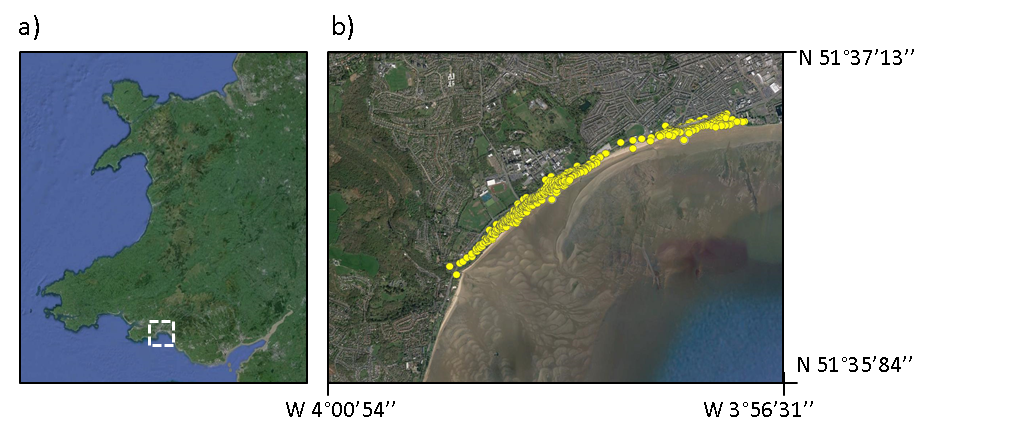


***Modelling airflows***

A digital elevation model of the observation site was built using terrain and building data. Terrain data were sourced from a LiDAR dataset with 2 m resolution from Geomatics-group [1]. The LiDAR data were converted from a projected (2D) coordinate system (UTM BNG), into a Geographic (3D) coordinate system (OSBG 36), before finally being converted into WGS 84 (lat, long) using the Petroleum transformation in ArcGIS (ArcGIS, 10.2, Esri). As the wind at the observation site was coming in over the sea, the elevation raster was edited to have the correct tidal height at the time the observations were made. Each tide height was calculated using sinusoidal interpolation between the recorded high and low tide heights The building data were generated using Ordinance Survey building plans and heights were taken from Digimap group [2, 3]. The terrain and building datasets were then combined and converted into the geographic coordinate system using ArcGIS.

Bird tracks were filtered to identify those where birds were exclusively gliding over the hotels along Swansea bay. These tracks were then transformed to the model coordinate system using the observers’ GPS position and North alignment. The model coordinate system used an origin with GPS position 51.610833°N, 3.958889°W, and rotation of 13^0^ clockwise from North, in order to align the mesh with the orientation of the majority of the buildings in the model. The mean radial distance from the bird to the buildings per run was calculated, as well as the angle between the bird and the buildings (figure S2).

Figure S2. The mean angle between the bird and the building is given by ϴ and the mean radial distance by R.


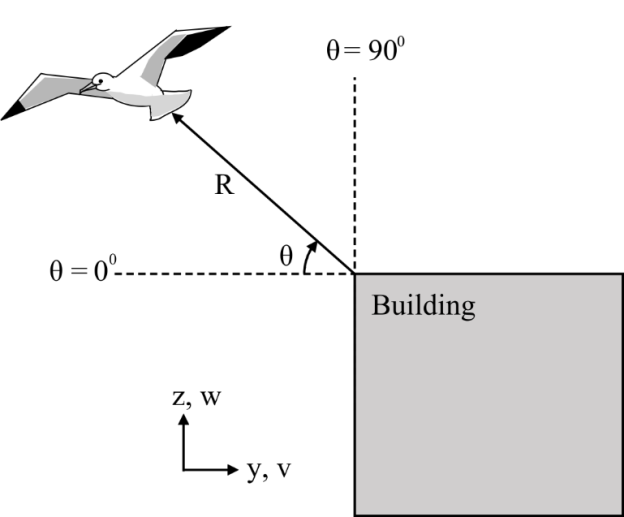


Wind field data were generated using the CFD model in the Quick Urban & Industrial Complex (QUIC) fast response dispersion modelling software, developed by Los Almos National Laboratory [4]. The QUIC-CFD model solves the 3D Reynolds-Averaged Navier-Stokes (RANS) equations using a simplified zero equation algebraic turbulence model, based on Prandtl’s mixing length theory using the fraction step method across the mesh [5]. The software is specifically designed to give relatively fast, yet accurate, wind fields in urban areas, and has been extensively validated against wind tunnel models and experimental urban wind field measurements [5]. The software has been shown to produce wind field estimates of a similar accuracy [6] to more computationally intensive approaches such as Large Eddy Simulation (LES). We used a 300 m x 300 m x 100 m [X x Y x Z] grid, with 1 m resolution, as the size of the mesh used was limited by the processing requirements.

The QUIC model was run to produce a wind field around the hotels, using the vertical wind profiles from the balloon releases as the input boundary conditions. These profiles of wind speed were smoothed to a best-fit logarithmic curve, and the wind direction was taken as the mean within each balloon ascent. Surface roughness and Monin-Obukhov reciprocal were taken as typical values for the terrain type [7, 8]. The surface roughness for the outer grid (open water) is 0.0002 and inner grid (open plain) is 0.005. The Monin-Obukhov reciprocal is negative during the day and a value of -0.0001 was used, as this gave the best qualitative match to the logarithmic wind profile.

The wind fields generated by the QUIC model were used in three ways. Firstly, to estimate the vector components of the airflow aligned to the model grid associated with the 3-dimensional position of the gulls in the observation fixes. Secondly to compare the vertical component of the airflow (the ‘w’ component) of the gull observation fixes with the maximum w available in the model. Lastly, to map the airspeeds that the gulls could theoretically use to fly along the hotels according to their position. For the latter, the average w values along the axis parallel to the hotel front were converted to estimates of flight speed using a flight model of equilibrium glide (figure S3) at a constant altitude, such that the glide sink rate is matched to the vertical air vector. The model was determined by a fixed-wing glide polar generated using the freeware ‘Flight’ [9] and the morphological measurements in the associated database. This produced a spatial velocity map of the feasible true airspeeds for simple orographic soaring. The validity of this flight dynamics model was then assessed by comparing the recorded ground speeds with predicted ground speeds. Predicted ground speeds were generated using the model wind components at the gulls’ average placement with respect to the buildings. The true airspeed was estimated at each location (as above) and then converted to ground speed by adding the cross-wind and supporting-wind vectors and assuming that the birds’ ground speed vector was parallel to the face of the hotels. This is demonstrated in figure S3, where there is some positive supporting wind vector (‘u’ component), which means that the gull’s ground speed is greater in the x direction than its true air speed. This generated two possible ground speed maps depending on the direction of flight. The average distance and angle of each flight path was then plotted over the feasible ground speed map, and compared with the individual’s recorded ground speed. This therefore provided a means to compare model predictions with empirical data.

Figure S3: a) Force vector diagram of a gull in equilibrium glide, where the lift and drag are equal to the body weight resolved into components using the glide angle as reference. b) A velocity vector diagram demonstrating ground speed and true airspeed of a gull flying in wind conditions consisting of a large cross wind and small supporting wind component.


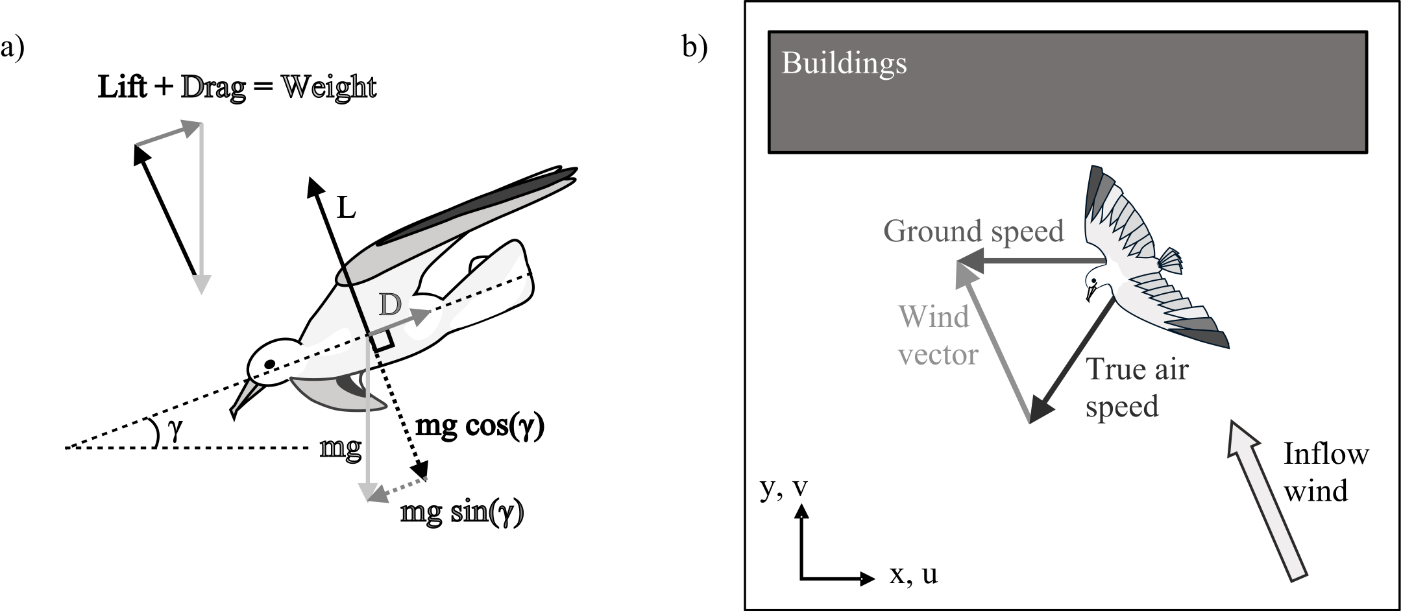


Comparison of the observed and predicted groundspeeds provided confidence in the map of feasible airspeeds, as the two speeds were positively correlated (Spearman’s correlation, n = 87, r_s_ = 0.56, *p* < 0.001). However, gulls were consistently flying faster than predicted by the model (Wilcoxon one-tailed signed ranks test, Z = 4.57, *p* < 0.001), which may be the result of an under-estimated wind field due to contemporary fluctuations in wind flow at the times of observation, or inaccuracies in the sink rate/air speed relationship defined by the birds’ glide polar.

***Statistical analysis***

*Space-use and flight type in relation to wind conditions*

Generalized additive models (GAMs) were used to analyse the effect of wind direction and strength on the number of gulls observed flying through the target area. Model fitting is summarised as follows: A ‘main effects plus interaction’ structure (using tensor product interactions) was used for the interaction between wind direction and strength. We used cubic regression splines except for wind direction, for which we used cyclic direction splines. We allowed for different functional forms of the effect of wind direction on the number of gliding birds above the line of hotels versus those with different flight trajectories, by fitting different smooth functions by factor level.

**Results**

***Comparison with theoretical glide polars***

Eighteen flight tracks (with 91 fixes overall) were collected from herring and lesser black-backed gulls gliding in apparently still air (a wind speed of 0 ms^-1^ was recorded with a handheld anemometer and a vertical smoke plume across Swansea bay confirmed that there was minimal change in wind speed with altitude). The minimum flight height for these tracks was 6 m above the substrate. Gulls glided at airspeeds between 9.5 and 17.2 ms^-1^ and their sink rates compared reasonably with theoretical predictions (figure S4). However, the range of values included sink rates close to 0 ms^-1^, suggesting that the air was not entirely still at the point of measurement (figure S4). We note that the theoretical glide polars of the two species overlap almost completely (figure S4). The airspeeds with the minimum sink rate are 6.8 and 6.4 ms^-1^ for the HG and LBB respectively, and the maximum glide ratios (in still air) occur at 10.5 and 10.0 ms^-1^ for the HG and LBB.

Figure S4. Theoretical glide polars for herring gulls (black line) and lesser black-backed gulls (grey line), as generated by ‘Flight 1.22’ for birds flying at sea level. Data recorded from birds gliding in still air are given as circles.

**
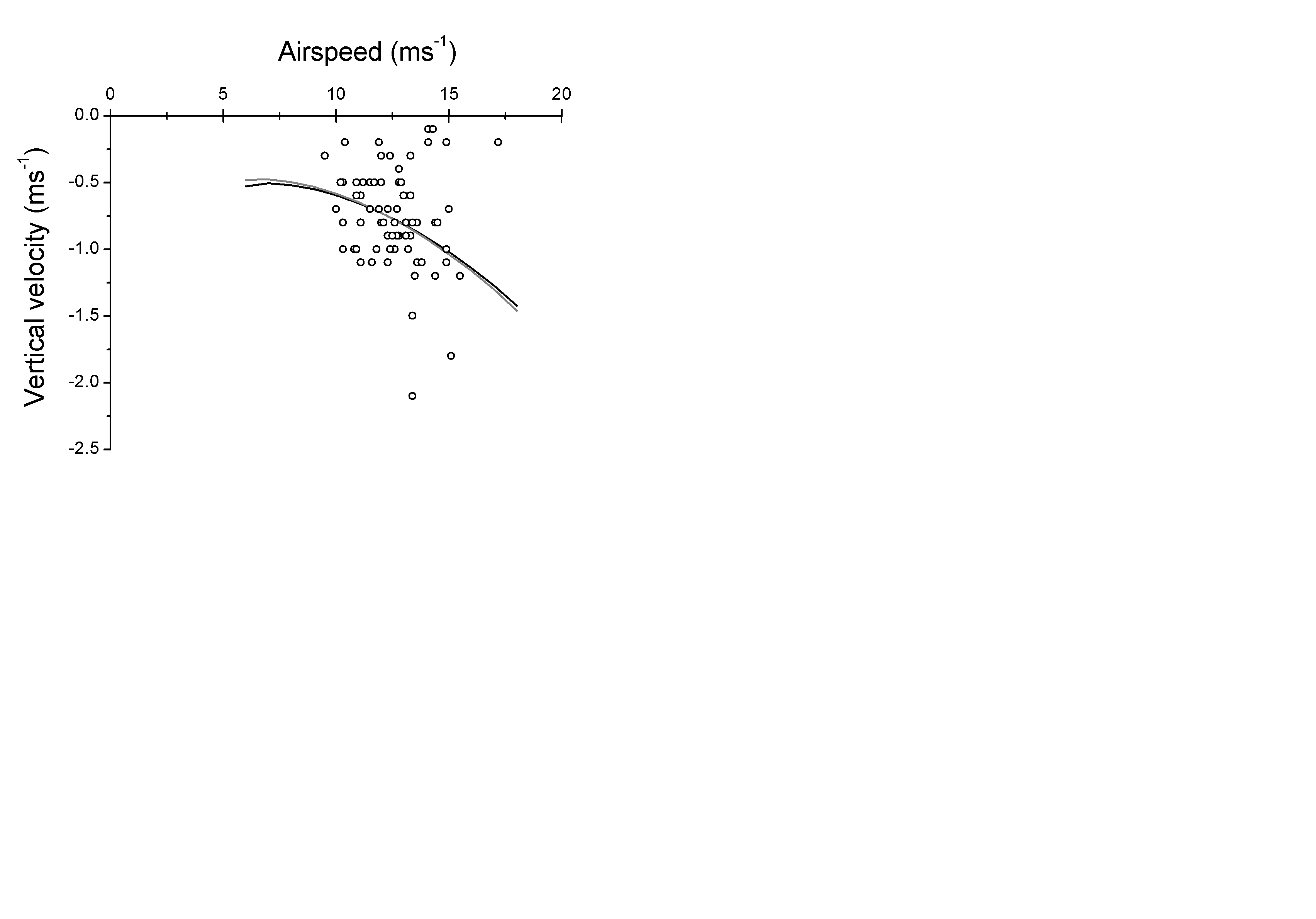
**

***Range of updraught speeds used by birds***

A total of 131 gull flight paths were used in the analysis of fine-scale position and airflow selection. The mean average w component selected by the gulls was 0.57 ms^-1^ (± 0.28 ms^-1^ s.d.). The maximum and minimum w component selected were 1.63 ms^-1^ and 0.05 ms^-1^ respectively. Examining the most commonly selected w component, 81% of the gulls flew in a range of between 0.4 ms^-1^ (just under the minimum sink in full extension glide) and 0.8 ms^-1^ (which according to Pennycuick’s flight model would relate to a 0.7 span reduction). The average w component is particularly interesting when looking at the gull glide polar, as 0.57 ms^-1^ is the sink rate associated with the best glide velocity (the flight speed related to the best glide angle).

**References**

[1] UK LIDAR DATA. Coverage: Ayr, Updated March 2014, Environmental Agency LIDAR, GB. Using: Geomatics-group data Service, <<http://geomatics-group.co.uk/>>, Downloaded: Feb 2015

[2] Digimaps (Street view and building heights). OS MasterMap Street view [GML geospatial data], Coverage: Ayr, Updated Oct 2014, Ordnance Survey, GB. Using: EDINA Digimap Ordnance Survey Service, <<http://edina.ac.uk/digimap>>, Downloaded: Feb 2015

[3] OS MasterMap Building Heights [GML geospatial data]. Coverage: Ayr, Updated Dec 2014, Ordnance Survey, GB. Using: EDINA Digimap Ordnance Survey Service, <<http://edina.ac.uk/digimap>>, Downloaded: Feb 2015. *OS MasterMap Building Heights [GML geospatial data]. Coverage: Ayr, Updated Dec 2014, Ordnance Survey, GB. Using: EDINA Digimap Ordnance Survey Service, <*[*http://edina.ac.uk/digimap*](http://edina.ac.uk/digimap)*>, Downloaded: Feb 2015*.

[4] Singh, B., Hansen, B.S., Brown, M.J. & Pardyjak, E.R. 2008 Evaluation of the QUIC-URB fast response urban wind model for a cubical building array and wide building street canyon. *Environ. Fluid Mech.* **8**, 281-312.

[5] Gowardhan, A.A., Pardyjak, E.R., Senocak, I. & Brown, M.J. 2011 A CFD-based wind solver for an urban fast response transport and dispersion model. *Environ. Fluid Mech.* **11**, 439-464.

[6] Neophytou, M., Gowardhan, A.A. & Brown, M.J. 2011 An inter-comparison of three urban wind models using Oklahoma City Joint Urban 2003 wind field measurements. *J. Wind Eng. Ind. Aerod.* **99**, 357-368.

[7] WMO. 1996 *Guide to meterological instruments and methods of observation*, Secretariat of the World Meteorological Organization.

[8] Obukhov, A.M. 1971 Turbulence in an atmosphere with a non-uniform temperature. *Bound.-Lay. Meteorol.* **2**, 7-29.

[9] Pennycuick, C.J. 2008 *Modelling the flying bird*. London, Elsevier.
